# Biological Characteristics of the Glacier Lantern Fish *Benthosema glaciale* (Myctophidae) in Icelandic Waters and the Irminger Sea During Summer

**DOI:** 10.1002/ece3.70386

**Published:** 2024-10-17

**Authors:** Charlotte S. Matthews, James Kennedy, Anna H. Ólafsdóttir, Christophe Pampoulie

**Affiliations:** ^1^ Marine and Freshwater Research Institute Hafnarfjörður Iceland; ^2^ University Centre of the Westfjords Ísafjörður Iceland; ^3^ Marine and Freshwater Research Institute Ísafjörður Iceland

**Keywords:** age structure, body condition, Iceland Basin, Irminger Sea, length–weight relationships, mesopelagic fish

## Abstract

Myctophids are one of the most diverse and abundant fish families in the mesopelagic zone, making them a key component in the marine pelagic community. In the North Atlantic, *Benthosema glaciale* (glacier lantern fish) is considered the most abundant myctophid north of 35° N, yet some regions within its extensive range lack information on its basic biological parameters. We investigated the growth and described the distribution of *B. glaciale* in Icelandic waters, including the southern continental shelf, Iceland Basin, and Irminger Sea. Length distribution, growth, relative body condition, age, and otolith‐fish size relationships were analyzed from samples collected opportunistically with pelagic trawls during the International Ecosystem Summer Survey in the Nordic Seas (IESSNS) in July 2020. In total, 1374 individuals were caught, and only a subset of 225 were measured fresh at sea and 89 were frozen and dissected on land. Total lengths of fresh fish ranged from 38 to 85 mm, while dissected individuals ranged from 40 to 74 mm and were 2 to 6 years old. Located over the Reykjanes Ridge in the Iceland Basin region, individuals had a significantly higher mean standard length and mean age, and a lower mean body condition. We reported length–weight relationships for both fresh and frozen samples, indicating evidence that preserving of the specimens through freezing affected relationships. The von Bertalanffy growth curve was calculated along with significant otolith‐fish size relationships where *r* ^*2*^ values ranged between 0.87 and 0.92. Our research highlights the importance of cross‐regional studies and provides baseline biology for *B. glaciale* in Icelandic waters, specifically in the southern continental slope and Reykjanes Ridge.

## Graphical abstract

In this study, we investigated the life history traits of an abundant myctophid, *Benthosema glaciale*, in Icelandic waters, and the Irminger Sea. Based on opportunistic sampling, we found significant differences in age structure and body condition between hydrographic regions.

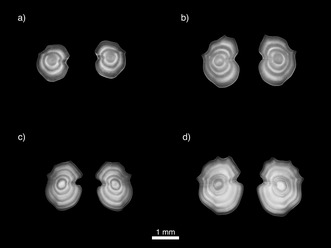

## 1 Introduction

Myctophids make up a high proportion of mesopelagic fishes as one of the most diverse and abundant fish families residing in the mesopelagic zone (200–1000 m; Catul, Gauns, and Karuppasamy 2011; Gjøsæter and Kawaguchi 1980). The global biomass of mesopelagic fishes has been estimated to be in the range of 10–15 billion tonnes (Irigoien et al. 2014; Proud et al. 2019). Given this high abundance, myctophids are a potential resource for commercial fisheries (Paoletti et al. 2021; Pauly et al. 2021; Vastenhoud, Bastardie, et al. 2023).

Mesopelagic fish typically occupy depths between 200 and 1000 m and display daily diel vertical migrations (DVMs, Bianchi et al. 2013; Catul, Gauns, and Karuppasamy 2011; St. John et al. 2016), whereby they migrate from the mesopelagic to the epipelagic layer during the night to feed. These migrations play a crucial role in the organic carbon transfer and sequestration through trophic interactions from the epipelagic to the bathypelagic layers (Martin et al. 2020). Myctophids commonly exhibit such migrations and, therefore, bridge the transfer of energy between lower and higher trophic levels through feeding upon organisms such as copepods, euphausiids and krill, and in turn, being an important prey for top predators, including marine mammals (Brophy, Murphy, and Rogan 2009; Pereira et al. 2011; Ringelstein et al. 2006), seabirds (Danielsen et al. 2010; Hedd et al. 2009; Thompson, Furness, and Monteiro 1998), and commercially important fishes, such as tuna, saithe, salmon, blue whiting, and mackerel (Battaglia et al. 2022; Hansen and Pethon 1985; Ólafsdóttir et al. 2016; Young et al. 2015). Such interactions from the surface to the deep waters highlight the importance that myctophids have in the marine food web and carbon cycle.

*Benthosema glaciale* is considered one of the most abundant and well‐studied myctophid species north of 35° N in the North Atlantic, with a broad distribution from Cape Verde off the west coast of Africa to the Mediterranean Sea and to subarctic waters (Badcock 1981; Gjøsæter 1981; Halliday 1970; Knutsen et al. 2023). Thus far, most of the studies on the ecology, age, growth and mortality of *B. glaciale* were conducted in other North Atlantic regions and adjacent seas, such as off the west coast of Canada (García‐Seoane, Dalpadado, and Vázquez 2013; García‐Seoane, Bernal, and Saborido‐Rey 2014; García‐Seoane et al. 2015; Halliday 1970; Kristoffersen and Salvanes 2009; Pepin 2013; Sameoto 1988) and the Norwegian Sea and Norwegian coastal waters (Dypvik, Røstad, and Kaartvedt 2012; Gjösæter 1973; Gjøsæter 1981; Suneetha and Salvanes 2001; Vastenhoud, Mildenberger, et al. 2023). Across the North Atlantic Ocean, the length distribution of *B. glaciale* greatly varied with smaller individuals (< 40 mm) dominating the Norwegian Basin, larger individuals (> 40 mm) dominating the Iceland Sea in the north of Iceland, and sizes mostly ranging from 30 to 50 mm in the Irminger and Labrador Seas (Knutsen et al. 2023). Those from the Flemish Cap in the northwest Atlantic were mainly between 50 and 65 mm (García‐Seoane et al. 2015).

Aging and demographic studies of *B. glaciale* have been conducted previously by counting the annuli on the otoliths (Albikovskaya and Rudneva 1986; Gjösæter 1973; Gjøsæter 1981; García‐Seoane et al. 2015; Halliday 1970), with individuals estimated to be up to 8 years old across its distribution range. Populations in different Norwegian fjords varied in their length–weight relationships and growth curves (Kristoffersen and Salvanes 2009), and also exhibited differences in the relationship between length and otolith diameter (Gjøsæter 1981). The feeding ecology of *B. glaciale* was also investigated (Pepin 2013; Knutsen et al. 2023) and revealed significant differences in prey selection and body condition across basins in the North Atlantic, including Iceland, Norwegian, Irminger, and Labrador Seas (Knutsen et al. 2023). This evidence of inter‐regional heterogeneity of populations makes regional studies essential for understanding the variability in this species' biology across its whole distribution range (Gjösæter 1973; Gjøsæter 1981; Knutsen et al. 2023), especially in regions where commercial or exploratory fishing activities have been initiated (Caiger, Lefebve, and Llopiz 2021).

Like other fishes, *B. glaciale* has been identified as prey by retrieval of otoliths from predator stomach contents (Battaglia et al. 2022; Danielsen et al. 2010; Hedd et al. 2009). Otoliths typically remain undigested in predators' stomachs as a result of their calcium carbonate structure. The often species‐specific shape of otoliths can therefore be used to identify and back calculate prey size as a result of previously determined relationships between otolith size and fish size (Campana 2005; Battaglia et al. 2010; Quigley et al. 2023). In various regions in the west and east North Atlantic, studies have reported these relationships for *B. glaciale* (Battaglia et al. 2015; García‐Seoane et al. 2015; Gjösæter 1973; Gjøsæter 1981; Halliday 1970).

Two deep sea basins are located south of Iceland. The Iceland Basin is 2000–3000 m deep and located approximately 30 km south of Iceland. This basin is defined by the Reykjanes Ridge in the west, the Faroe Islands‐Iceland Ridge in the north, and Rockall Bank in the east. The Irminger Sea is a 3000–4000 m deep basin located west of Iceland, between the east coast of Greenland and the Reykjanes Ridge. The warm North Atlantic current flows towards both basins, on both sides of the Reykjanes ridge, leading to differences in their hydrographic characteristics (Petit, Mercier, and Thierry 2021), that is, temperature, salinity, and consequently water masses densities (Sarafanov et al. 2007). The Icelandic basin is generally more saline and warmer than the Irminger Sea (Sarafanov et al. 2007; Sutton and Sigurðsson 2008). Several studies have reported a continuous deep scattering layer (DSL) with varying densities and abundance of mesopelagic organisms within and between the Irminger Sea and the Iceland Basin (Magnússon 1996; Sigurðsson, Jónsson, and Pálsson 2002). Sigurðsson, Jónsson, and Pálsson (2002) noted that the depth of the DSL varied from 200 to 800 m at night. *B. glaciale* has been described as one of the most abundant species in the DSL both in the Irminger Sea and the Iceland Basin (Dolgov 2015; Fock and John 2006; Magnússon 1996; Sigurðsson, Jónsson, and Pálsson 2002; Sutton and Sigurðsson 2008); yet, information on its distribution, age structure, length–weight relationships, body condition, and otolith‐fish size relationships is limited, specifically for the Iceland Basin and continental shelf area.

In the current study, we used opportunistic sampling of *B. glaciale* during the International Ecosystem Summer Survey in the Nordic Seas (IESSNS) conducted in July 2020 to investigate biological parameters such as length and age distributions, length at age relationships, body condition, and growth rate. The parameters were compared between samples collected in different hydrographic regions to the south of Iceland, in the Iceland Basin and Irminger Sea. Furthermore, we research the relationship between otolith size and fish size to provide vital information to convert otoliths sampled from predators into mesopelagic prey species and prey size range.

The main objective of this study was to gather more information on *B. glaciale* in Icelandic waters with a specific interest in condition, length, and age distribution across basins.

## 2 Materials And Methods

### 2.1 Sample Collection

**FIGURE 1:**
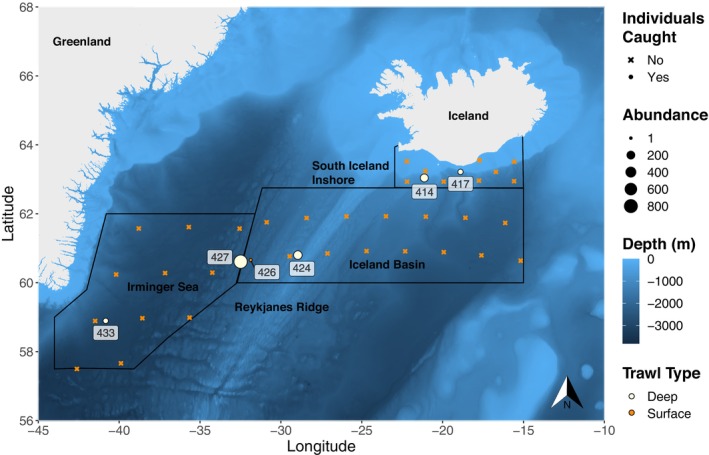
Total abundance per station of *Benthosema glaciale* caught during the IESSNS in July 2020 at deep and surface trawls. Station number where individuals were caught is displayed and stations with no catch for the species are also displayed. Thick black lines show the strata boundaries.

*Benthosema glaciale* were collected and frozen during the Icelandic part of the IESSNS from July 11th to 30th, 2020 (Ólafsdóttir and Kennedy 2020) aboard the research vessel Árni Friðriksson. The IESSNS is an extensive pelagic trawl survey aimed at quantifying the abundance and distribution of commercially important pelagic species; Atlantic mackerel (*Scomber scombrus*), Atlantic herring (*Clupea harengus*), and blue whiting (*Micromesistius poutassou*). Survey sampling locations were randomly stratified according to IESSNS protocols (Nøttestad et al. 2016). Samples were collected in the Irminger Sea (to the west of the Reykjanes Ridge), Iceland Basin (including the Reykjanes Ridge and continuing eastward), and south Iceland inshore (to the east of the Reykjanes Ridge on the continental shelf and slope, Figure 1). The Multpelt 832 pelagic trawl net used during sampling was designed for targeting larger, fast‐swimming pelagic fishes, with a cod‐end mesh size of 50 mm, therefore other species were considered opportunistic bycatch. Two trawl methods were used: (1) predetermined standardized surface trawls (termed surface trawl) conducted close to the surface for 30 min at a speed of 5 knots with the head rope visible at the surface and footrope at approximately 30–35 m depth; and (2) opportunistic trawls targeting acoustic registrations identified as blue whiting at depths ranging from 50 to 500 m (termed deep trawl; Nøttestad et al. 2016) with a trawling time of *circa* 60 min and a speed of *circa* 3.5 knots. The gear specification can be found in Ólafsdóttir and Pampoulie (2020). Individuals were identified and counted on board at each trawl station. Out of the 1374 individuals caught, a random subset of 225 individuals collected from multiple trawl stations total wet weight (to the nearest gram, g) and total length (to the nearest millimeter, mm) were measured on board, and were frozen for later processing. The fishes were not frozen individually and out of these frozen individuals, only 89 were intact (not badly damaged) when processed for dissection post‐freezing.

### 2.2 Length, Weight, and Relative Condition

Individuals were dissected on land and weighed (frozen, thawed, and gutted weight), to the nearest 0.1 g. Post thawing at room temperature, total length (TL, snout to caudal fin tip), fork length (FL, snout to shortest caudal fin ray), and standard length (SL, snout to bone knot in the caudal peduncle; Jennings, Kaiser, and Reynolds 2001) were measured to the nearest 0.1 mm. For length and weight analyses of frozen specimens, the weight variable used was gutted weight (*W*_g_), to remove variability resulting from stomach contents (Froese 2006). Due to tail damage sustained from sampling and freezing impacting total and fork length measurements, SL was used as the length variable for analyses of frozen specimens. During the survey, 225 specimens had TL and total wet weight measured while freshly caught, with three were excluded from analyses due to measurement errors (final *n* = 222). To compare measurements from freshly caught versus frozen individuals, TL was used as this was the only length measurement recorded at sea and total weight (wet weight fresh at sea and thawed weight from frozen specimens). Of the 89 frozen individuals, 86 were used in this comparison as three fish had extensive damage to their caudal fin, resulting in outliers for TL measurements. The length–weight relationship (LWR) was determined using the natural logarithm transformed linear model ln(*W*) = ln(*a*) + *b* × ln(*L*), converted to the power equation *W* = *a* × *L* ^*b*^, where *a* is the intercept and *b* is the regression coefficient (Le Cren 1951). To examine the body condition of *B. glaciale*, a relative condition factor (*K*_*rel*_) was calculated based on the ratio between the gutted weight of the individual (*W*_g_) and the estimated weight from the pooled LWR (Czudaj, Möllmann, and Fock 2022; Le Cren 1951; López‐Pérez et al. 2020). To compare the calculated LWR based on dissected samples versus those measured on board, TL measurements were used with total wet weight (measured on survey) and total thawed weight (measured during dissections).

### 2.3 Otolith Morphometrics and Aging

Sagittal otoliths were removed from each individual, cleaned with water, and stored in labeled, dry sampling trays for imaging. Images of otoliths were taken on a matte background with and without emersion in water, using the integrated Leica IC90 E camera within the Leica MZ6 stereomicroscope at a magnification of 2.0×. For morphometric measurements, images of otoliths without emersion were used. Otolith morphometric measurements including otolith length (mm), area (mm^2^), and width (mm) were measured to the nearest 0.001 mm using ImageJ software via the Analyze Particles function, after images were thresholded and converted into an 8‐bit binary file (Schindelin et al. 2012). Left otoliths were used consistently for measurements.

Fish were aged by counting annual increments defined by alternating opaque and translucent bands from images of otoliths. The left otolith was immersed in water with the proximal side down, and viewed under a Leica M165 C stereomicroscope with an integrated camera, using reflected light and a black background. One year of growth was defined as including one opaque and one translucent ring, based on seasonal variation expected to impact growth (Campana 2001). Annual rings of the otoliths were counted by two independent readers, and then if there were any inconsistencies in aging between them, both readers would continue to examine them until reaching agreement, or the otolith was excluded from the analysis. From the frozen specimens (*n* = 89), we could only retrieve 88 otolith pairs for their morphometrics and age assessment.

### 2.4 Data Analysis

Due to the low number of individuals collected per station, individuals were pooled by region for analyses (Irminger Sea: two stations, Iceland Basin: two stations, and south Iceland inshore two stations). Statistical analyses and figures were conducted and created with R statistical software (version 4.2.2 “Innocent and Trusting”; R Core Team 2022). The assumption of data normality was examined by visually assessing quantile–quantile plots and conducting a Shapiro–Wilke test (Shapiro and Wilk 1965). Equal variances were evaluated using a Levene's test (Levene 1960). Variables were natural logarithm transformed (ln) to meet normality and variance assumptions when necessary. Mean SL, age, and *K*_rel_ were compared between regions by analysis of variance (ANOVA) or by Kruskal–Wallis test if normality or equal variance assumptions were violated. To test whether the LWR conveyed allometric or isometric growth, a student's *t*‐test was used to determine if *b* = 3 (isometric) or *b* > 3 (positive allometric) or *b* < 3 (negative allometric; Froese, Tsikliras, and Stergiou 2011; Ogle 2022). The growth curve of *B. glaciale* was calculated by fitting a von Bertalanffy growth model to the measured SL from individuals that were aged with the function: *L*_*t*_ = *L*_∞_ [1–*e* ^(−*k*(*t–t*0))^] where *L*_*t*_ is the average SL at age *t*, *L*_∞_ is the asymptote length based on the model of the average length at age, *k* is the growth rate coefficient and *t*_0_ is the age when the average SL based on the model is zero (Cailliet et al. 2006; von Bertalanffy 1938). Estimates were calculated using the FSA Simple Fisheries Stock Assessment package (Ogle et al. 2023). Bootstrapped 95% confidence intervals for the growth model were calculated. Due to the non‐linearity of the relationships between otolith characteristics (length, area, and width) and fish size measurements (SL and *W*_g_), the data were analyzed by ln transformed linear regressions, and the strength of correlation was compared using the adjusted coefficient of determination (*r* ^*2*^).

## 3 Results

A total of 1374 *B. glaciale* (Figure 1) were caught in the Iceland Basin, Irminger Sea, and south Iceland inshore at five deep trawls ranging from 200 to 550 m in depth and one surface trawl station (Figure 1). The trawl station located west of Reykjanes Ridge in the Irminger Sea (427) had the highest abundance of 823 individuals (Figure 1). Only one individual was caught at the surface trawl station (426) adjacent to station 427, but based on the defined sampling regions, it was caught within the Iceland Basin region.

### 3.1 Length and Weight Relationships

**FIGURE 2:**
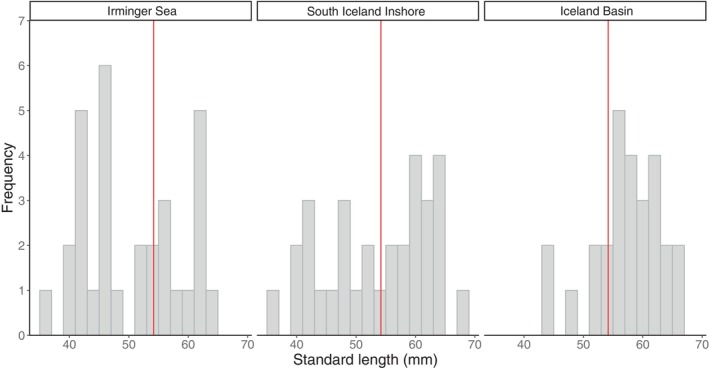
Frequency distributions of the standard length (mm) (red line: Total sample mean = 54.2 mm) of *Benthosema glaciale* collected during IESSNS in July 2020 in three separate regions (bar width = 2 mm).

**FIGURE 3:**
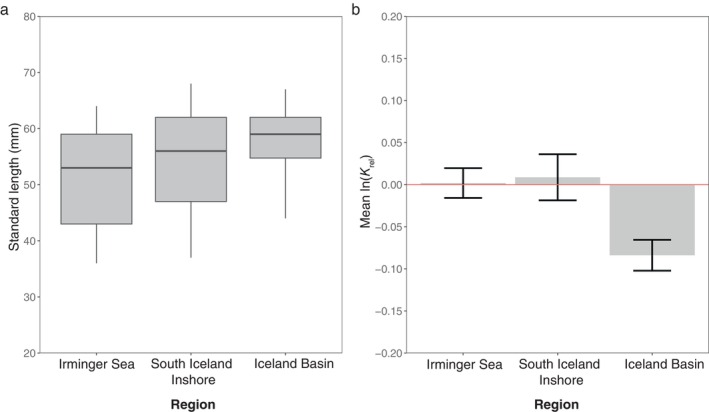
Comparisons of (a) the standard length (mm) of *Benthosema glaciale* by boxplot with the thick black line representing the median value within the gray boxes extending from the 25th (bottom) to 75th (top) percentiles with whisker bars showing the maximum range of adjacent values and (b) the positive and negative values of the natural logarithm transformed mean relative body condition (*K*_rel_) shown as the gray bar with standard error bars of individuals collected from three regions during IESSNS in July 2020. The red line is the zero axis of the mean ln(*K*_rel_).

Individuals collected from five trawl stations (414, 417, 424, 426 and 427; Figure 1) in the south Iceland inshore (*n* = 31), Iceland Basin (*n* = 27), and Irminger Sea (*n* = 31) were examined for SL−*W*_g_ comparisons. The SL of dissected individuals ranged from 36 to 68 mm (Figure 2). Though ranges were similar across basins, there were significant regional differences in SL (Kruskal‐Wallis test: *H*_(2)_ = 8.62, *p* < 0.05, *n* = 89, Figures 2 and 3), with Irminger Sea having a significantly lower mean than Iceland Basin (Wilcoxon rank sum test: *W* = 226.5, *p* < 0.01, *n* = 58, Figure 3a). There was no significant difference in mean SL between the south Iceland inshore and Iceland Basin (Wilcoxon rank sum test: *W* = 330, *p* = 0.170, *n* = 58) or the Irminger Sea and south Iceland inshore (Wilcoxon rank sum test: *W* = 377, *p* = 0.147, *n* = 62, Figure 3a).

**FIGURE 4:**
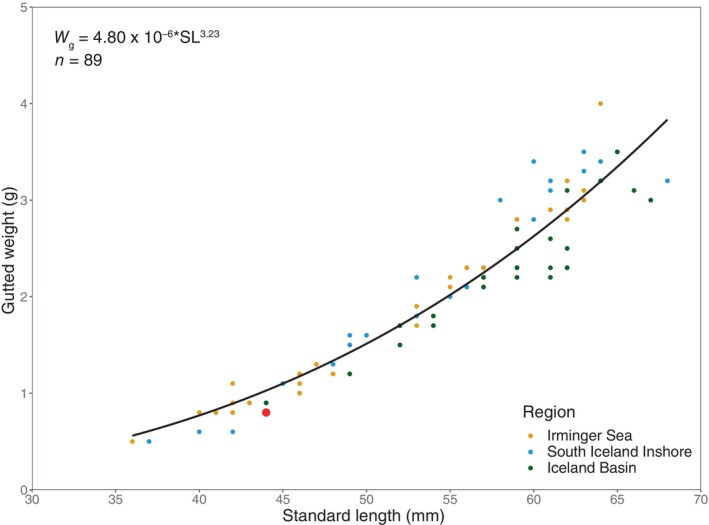
Standard length‐gutted weight relationship for frozen *Benthosema glaciale* individuals, collected from three regions during the IESSNS in July 2020. The red circle indicates one individual collected from a surface trawl station (426) in the Iceland Basin region.

The ln transformed *K*_rel_ of individuals collected from the Iceland Basin was significantly lower than both Irminger Sea (student's *t*‐test: *t*_(55.35)_ = 3.367, *p* < 0.01, *n* = 58, Figure 3b) and south Iceland inshore individuals (student's *t*‐test: *t*_(51.1)_ = 2.809, *p* < 0.01, *n* = 58). Between the Irminger Sea and south Iceland inshore, *K*_rel_ did not significantly differ (student's *t*‐test: *t*_(51.3)_ = −0.209, *p* = 0.835, *n* = 62). Combining data from the three areas, the logarithm transformed linear length–weight model resulted in a *b* value significantly > 3 (student's *t*‐test: *t*_(87)_ = 2.966 *p* < 0.01; *b* = 3.23; 95% Confidence Interval: 3.08–3.38, *n* = 89) indicating positive allometric growth (Figure 4).

### 3.2 Comparing Sampling Types

**FIGURE 5:**
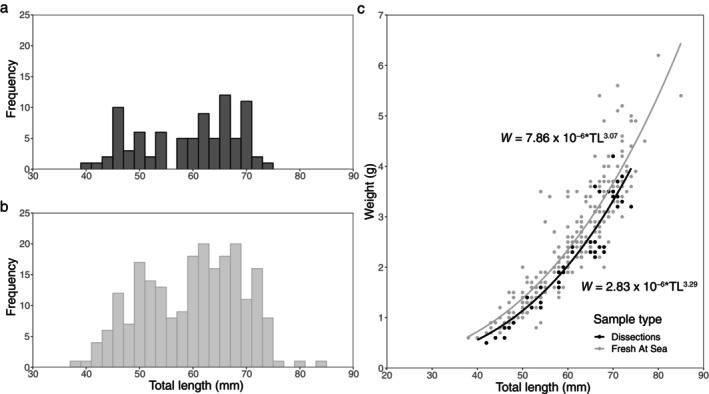
Total length (mm) frequency distribution of (a) 86 dissected individuals post‐freezing and (b) 222 individuals measured fresh at sea of *Benthosema glaciale* collected during the IESSNS in July 2020. (c) The total length (TL)–total thawed weight (*W*) relationship of individuals measured during dissections post‐freezing (black) and those measured fresh at sea (gray).

The pooled dissected samples' TL frequency distribution showed a distribution with a constricted range and peaks at smaller lengths compared to the TL distribution of individuals measured fresh at sea (Figure 5a,b). The apparent bimodality present in the length distribution of the dissected individuals was also evident in the fresh samples. The TLs of the fresh and dissected samples ranged from 38 to 85 and 40 to 74 mm, respectively. The calculated LWR from the fresh individuals resulted in *b* = 3.07 with a 95% confidence interval between 2.91 and 3.23, which was low compared to the value of the LWR using TL (*n* = 222) and thawed weight available from the dissection data post‐freezing (*n* = 86) where *b* = 3.29 with the 95% confidence interval between 3.13 and 3.45 (Figure 5c). Previous studies showed variation in regression coefficient values (*b*) of *B. glaciale* from LWR calculations across regions in the North Atlantic (Table 1).

**TABLE 1:** Regression coefficient values (*b*) from length–weight relationships determined from individuals sampled in regions in the North Atlantic with the standard length (SL) range and sample size (*n*). Adapted from Badouvas, Somarakis, and Tsagarakis (2022).

Region	Length range (mm)	*b*	*n*	Specimen preservation	Sampling period
North Atlantic (Fock and Ehrich 2010)	21–81	3.020	21	Unknown	Multi‐year, May–June
Tropical Atlantic (López‐Pérez et al. 2020)	15–35	3.647	15	Not preserved (fresh)	Single year, April
West Mediterranean (Olivar, Molí, and Bernal 2013)	14–47	3.093	249	Formalin	Multi‐year, multi season
East Mediterranean (Badouvas, Somarakis, and Tsagarakis 2022)	20–74	2.916	691	Frozen	Single year, multi season
Western Atlantic (Sameoto 1988)	15–80	3.090	553	Formalin and seawater	Multi‐year, multi season
Upper North Atlantic (Knutsen et al. 2023)	23–75	3.287	230	Back calculated from ethanol to fresh values	Single year, May–June
Present study	36–68	3.230	89	Frozen	Single year, July

### 3.3 Age Reading From Otoliths

**FIGURE 6:**
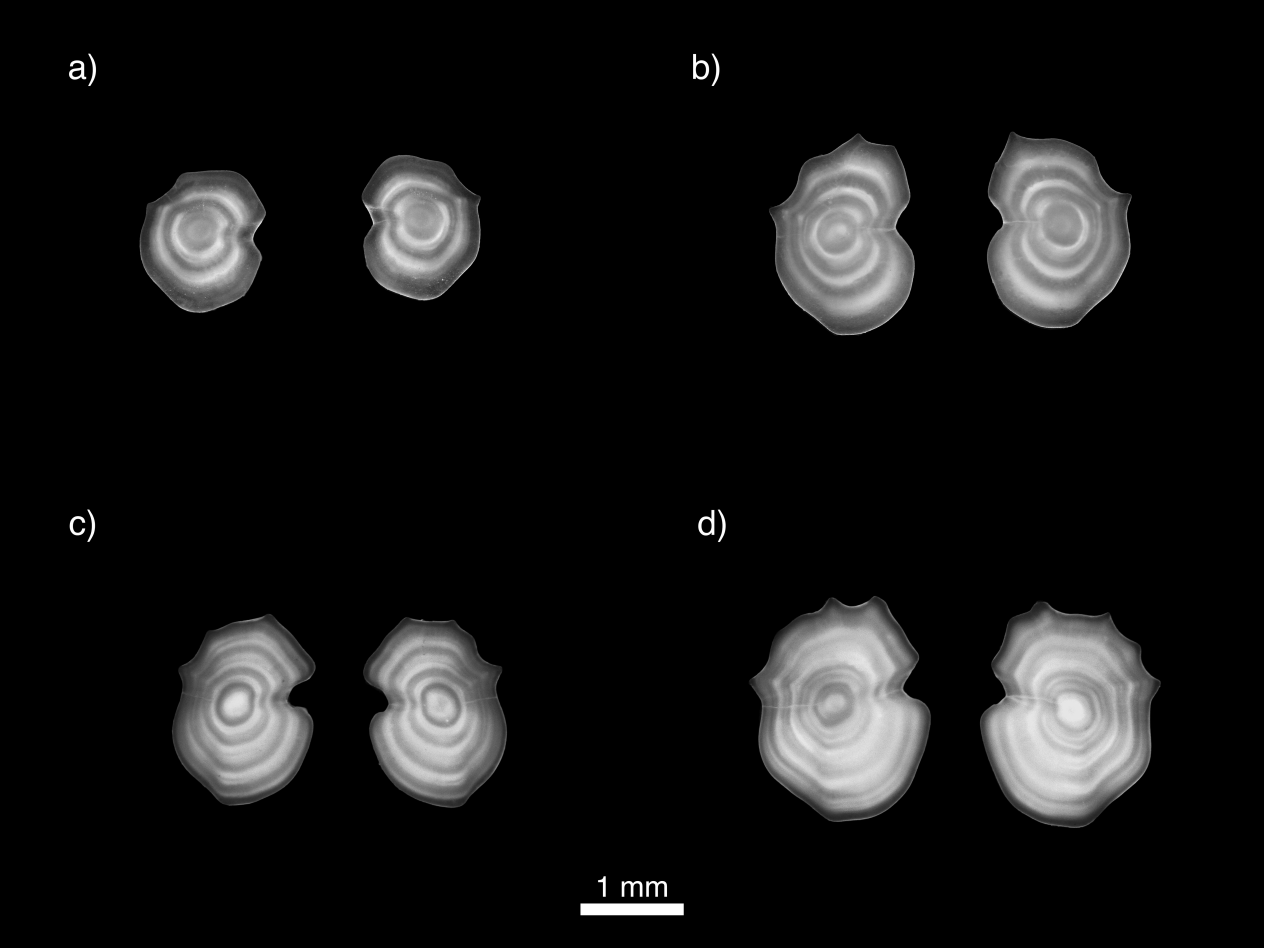
Left and right otoliths of *Benthosema glaciale* with the proximal side down, collected during the IESSNS in July 2020 (seasonal growth period) with a developing opaque edge: (a) 2 years old, (b) 3 years old, (c) 4 years old, and (d) 5 years old.

**FIGURE 7:**
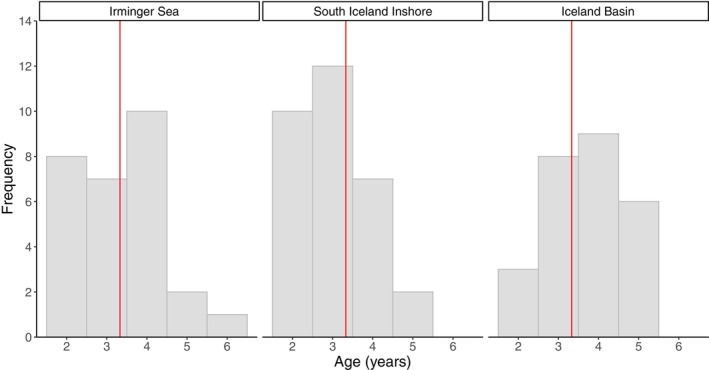
Frequency distributions of the age in years of *Benthosema glaciale* collected during IESSNS in July 2020 in three separate regions (red line: Total sample mean = 3.3).

**FIGURE 8:**
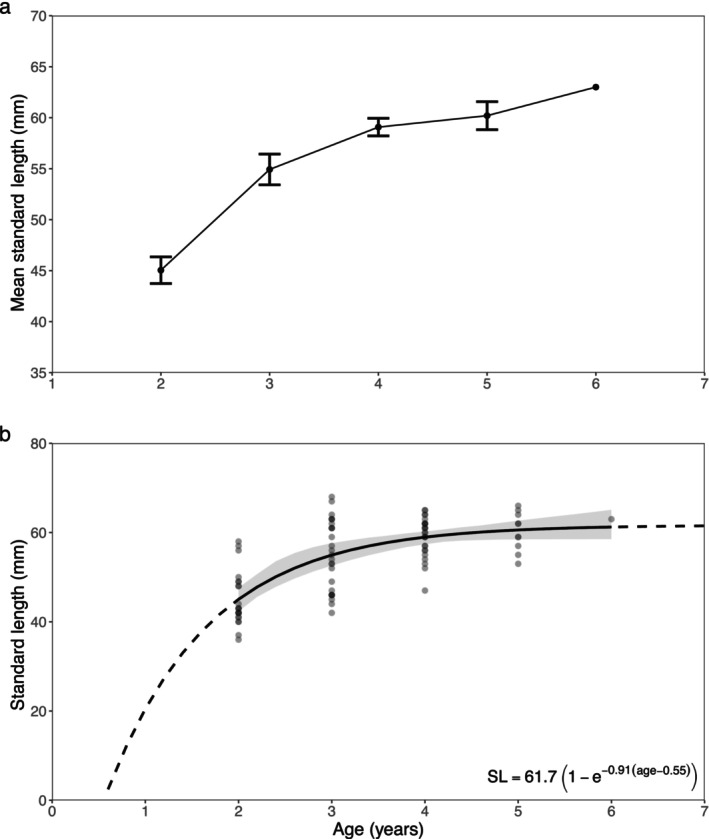
(a) Mean standard length (mm) for each age group (years) of *Benthosema glaciale* collected during the IESSNS in July 2020. Error bars denote the standard error of the mean for each age. (b) Von Bertalanffy growth curve with bootstrapped 95% confidence intervals (gray shading). Dotted line depicts the estimated curve outside the range of the observed datapoints; dots depict values for each individual.

Out of the 88 sets of otoliths, only 86 could be aged and agreed upon. Based on otolith annuli (Figure 6), individuals age ranged from 2 to 6 years old, with ages 3 to 4 making up 61.6% of the samples. In the Irminger Sea, individuals were between 2 and 6 years old, with a mean age of 3.3 (*n* = 29, standard deviation [SD] = 1.1), with the majority being 2–4 years old (89.6%, Figures 7 and 8). In the Iceland Basin region, individuals were between 2 and 5 years old with a mean age of 3.7 (*n* = 26, SD = 1.0), of which were mostly 3–4 year olds (65%) and a few age 2. Along the continental shelf and slope in the inshore area, individuals were between 2 and 5 years old as well, with a mean age of 3.0 (*n* = 31, SD = 0.9), mainly comprised of 2–3 year olds (71.0%). When comparing mean age between regions, individuals from the Iceland Basin were significantly older than the ones sampled in the other two regions (Kruskal–Wallis test: *H*_(2)_ = 6.132, *p* < 0.05, Figure 7, *n* = 86). The mean age did not differ significantly between the Irminger Sea and south Iceland inshore samples (Wilcoxon rank sum test: *W* = 503, *p* = 0.412, *n* = 55). The SL of 2‐year‐olds ranged from 36 to 58 mm (*n* = 22), 3‐year‐olds ranged from 42 to 68 mm (*n* = 27), 4‐year‐olds were 47–65 mm (*n* = 26), 5‐year‐olds were 53–66 mm (*n* = 10), and one age six individual was 63 mm (Figure 8a). The calculated von Bertalanffy parameters for the 86 individuals were *k* = 0.91 (95% Confidence Interval: 0.32–2.00), *L*_∞_ = 61.68 (58.0–72.8), and *t*_0_ = 0.55 (−1.19–1.29, Figure 8b).

### 3.4 Otolith‐Fish Size Relationships

Otoliths from *B. glaciale* individuals (*n* = 88) exhibited a mean length of 2.1 mm (SD = 0.3), mean area of 2.5 mm^2^ (SD = 0.6) and a mean width of 1.6 mm (SD = 0.2). Adjusted *r* ^*2*^ of the relationships between otolith measurements and fish size ranged from 0.87 to 0.92, the lowest being *W*_g_ versus otolith width, and the highest being SL versus otolith area (Table 2). All regressions between the fish length and the otolith measured were significant for each variable tested (Table 2).

**TABLE 2:** Linear regression model equations of standard length (SL) and gutted weight (*W*_g_) to otolith length (OL), otolith area and otolith width (OW) of *Benthosema glaciale* individuals collected from frozen individuals during the IESSNS in July 2020 (*n* = 88).

Regression	Equation	Adjusted *r* ^*2*^	*p*
SL vs. OL	$\ln\left(O_{L}\right)=0.87\times \ln\left(\mathrm{SL}\right)-2.75$	0.89	< 0.001
*W* vs. OL	$\ln\left(O_{L}\right)=0.26\times \ln\left(W\right)+0.57$	0.89	< 0.001
SL vs. OA	$\ln\left(O_{A}\right)=1.65\times \ln\left(\mathrm{SL}\right)-5.72$	0.92	< 0.001
*W* vs. OA	$\ln\left(O_{A}\right)=0.50\times \ln\left(W\right)+0.56$	0.91	< 0.001
SL vs. OW	$\ln\left(O_{W}\right)=0.86\times \ln\left(\mathrm{SL}\right)-2.99$	0.90	< 0.001
*W* vs. OW	$\ln\left(O_{W}\right)=0.26\times \ln\left(W\right)+0.28$	0.87	< 0.001

## 4 Discussion

We present biological parameters of *B. glaciale* from three different oceanographic regions: the Irminger Sea, the Icelandic Basin, and the south Iceland inshore. Although our approach was based on a one‐year sampling, cross‐regional comparison of samples indicated differences in length distribution, body condition, and age of *B. glaciale*. While one of the most studied mesopelagic species in the North Atlantic (Knutsen et al. 2023), data on *B. glaciale* are lacking in the Icelandic waters, specifically in the Iceland Basin and southern continental shelf. The observed distribution of *B. glaciale* seems to overlap with previous records from targeted mesopelagic surveys conducted in the summers of 1996, 1999, and 2001 (Sigurðsson, Jónsson, and Pálsson 2002).

Our study also revealed regional variation in the size distribution and age of *B. glaciale*. Since the same gear was used during the sampling, these results are potentially indicative of ontogenetic migrations and life history variability between basins. It is, however, important to note that conclusions were based on a limited number of deep trawl stations. Although collected in the defined Iceland Basin region, the station where individuals were sampled in this area was located over the Reykjanes Ridge. To the west of the Reykjanes Ridge in the Irminger Sea, individuals sampled were, on average, smaller, and younger than those collected in the Iceland Basin region. *B. glaciale* from higher latitudes in the North Atlantic usually mature at around 2 to 3 years old (García‐Seoane et al. 2015; Gjøsæter 1981), suggesting that the majority of individuals in the current study may be mature. Compared to the minimum and maximum age distribution found in prior studies in the northwest Atlantic (1 to 7 years; García‐Seoane et al. 2015) and northeast Atlantic (1 to 8 years; Gjøsæter 1981), the age range was narrower in the present study with individuals between 2 and 6 years old. Due to a sampling protocol designed for larger fishes, the mesh size of the trawl used was 50 mm and therefore smaller, younger individuals (< 2 years) were not caught, which could be due to gear selectivity. Therefore, the present result might not be directly comparable to previous studies when it comes to age distribution.

Prior studies indicate spatial variations in the length distribution of *B. glaciale* across regions in the North Atlantic (García‐Seoane et al. 2015; Knutsen et al. 2023). Individuals analyzed from the Irminger Sea within our study were caught in the northeastern area of the basin and had a higher frequency of larger individuals, whereas Knutsen et al. (2023) caught individuals with peak relative length frequency at 40 mm in more southern areas, which may explain the observed differences. Sampling design, sample size, as well as gear selectivity might also be another plausible explanation for the observed differences. The variation in length distribution and condition, that is, growth pattern, could also be interpreted as an indication of temperature differences (Baudron et al. 2014) or prey availability (Jacobson et al. 2018) between the ocean basins around Iceland (Petit, Mercier, and Thierry 2021; Sarafanov et al. 2007). Evidence of decreasing prey biomass (zooplankton) and increasing sea temperature from the Irminger Sea towards the Iceland Basin may be contributing factors to these across basin differences (Sutton and Sigurðsson 2008; Gíslason et al. 2021). Although a rise in temperature can improve growth performance in marine fish (dos Santos Schmidt et al. 2020; Neuheimer et al. 2011), surpassing the optimal range will cause an increase in metabolic costs and will often lead to a smaller body size to reduce the risk of oxygen deprivation (Baudron et al. 2014; Daufresne, Lengfellner, and Sommer 2009; Neuheimer et al. 2011). Similarly, an increase in prey size (Jacobson et al. 2018), quality (Knutsen et al. 2023), and biomass (Ofelio et al. 2023) usually leads to optimal growth patterns in marine fishes, although the combined effect of both temperature and prey characteristics are intertwined (Arula et al. 2015; dos Santos Schmidt et al. 2020; Ofelio et al. 2023). Our combined length and age data suggest that the observed difference among *B. glaciale* samples appears to be influenced by the uneven distribution of the different age cohorts in each region.

Significant differences in the relative condition of individuals were found in samples from different hydrographic regions. In Iceland Basin, individuals had a lower body condition while also being relatively older than those from other regions. In previous findings, larger individuals collected north in the Iceland Sea had a relatively higher body condition than smaller individuals examined from other ocean basins (Knutsen et al. 2023). These high body condition individuals had full stomachs and were positively selecting for a lipid rich prey species, which may be contributing to this positive condition (Knutsen et al. 2023). Stomach contents and fullness were not examined within our study, but this, as well as maturity, may be an important element for future research.

Knowledge of the factors related to maturity and reproduction is lacking for *B. glaciale* in Icelandic waters, and this is a possible direction for future studies as this was not examined in the present study. The timing and locations of spawning of *B. glaciale* are unknown in this region. In the Northwest Atlantic, *B. glaciale* has been found to spawn in the early months of the year (January to April) and in the Northeast Atlantic in the summer months (June to July; García‐Seoane, Bernal, and Saborido‐Rey 2014; Gjøsæter 1981; Knutsen et al. 2023). Sexual differences in growth have not been observed for *B. glaciale* (García‐Seoane et al. 2015), therefore any differences in the sex ratio between regions are unlikely to be major factor in explaining the regional differences in biological parameters uncovered during the present study.

The positive allometric growth we detected with the LWR indicates faster growth in weight than length (López‐Pérez et al. 2020). Positive allometric growth has been suggested to be an important characteristic for diel vertical migrations, and is common among myctophid species which requires robust muscles for energetically taxing large‐scale movement (Badouvas, Somarakis, and Tsagarakis 2022; López‐Pérez et al. 2020; Olivar, Molí, and Bernal 2013). Our study shows a higher degree of allometric growth compared to *B. glaciale* samples collected in 1983–85 in the North Atlantic along the mid‐Atlantic ridge between 43° and 59° North (*b* = 3.020; Fock and Ehrich 2010), as well as other studies from lower latitudes (except the tropical Atlantic, Table 1; Badouvas, Somarakis, and Tsagarakis 2022). This could be due to an intraspecific selection process driving *B. glaciale* individuals to gain weight faster with length at higher latitudes, potentially caused by differences in temperature and seasonality. However, comparisons across the North Atlantic are limited due to variation in sampling methods with occurrences of both single year and multi‐year studies, as well as studies that covered multiple or single seasons, and used different gears. Depending on the season and reproduction, LWR can be influenced by factors related to maturity and feeding as well as environmental conditions that may change over the year, impacting growth (Czudaj, Möllmann, and Fock 2022; Froese 2006). For example, our study is limited to samples collected during a single month (July) of a specific year in higher latitudes of the North Atlantic, which influences the calculated LWR. Studies that are conducted over multiple seasons may provide a more general LWR. In contrast, samples collected during periods of increased feeding activity and fast growth could potentially have a higher allometric value than those post‐spawning, where individuals have allotted resources and energy to reproduction (Brosset et al. 2016; Le Cren 1951).

The preservation method of specimens used in LWR analyses can also affect the estimated growth parameters (Ajah and Nunoo 2003; Anzueto‐Calvo et al. 2017; Ogle 2009). As shown in Table 1, studies of LWR for *B. glaciale* have used different preservation methods, including freezing, ethanol, formalin, and no preservation. Freezing, formalin, and ethanol are common preservation techniques in biology that have been found to have different levels of effect on LWR of fishes due to processes such as shrinking of tissues (Ajah and Nunoo 2003). Preservation techniques such as formalin and ethanol result in a higher *b* value and decreased body condition factor when examining the LWR (Anzueto‐Calvo et al. 2017). The length‐distributions comparing fresh and frozen samples in our study showed that shrinkage of specimens occurred through freezing with a shift and constriction of length range, and the *b* value was higher after freezing as well, similar to the findings of Anzueto‐Calvo et al. (2017). Our comparisons between fresh and frozen samples were limited because individual fish could not be compared pre‐ and post‐preservation and a calibration equation could not be calculated. A subsample of labeled individuals should be analyzed in future studies to calculate these values.

From the total sample of dissected individuals (*n* = 86), the total length frequency distribution showed less distinct modes in comparison to the distribution of those that were measured fresh during the survey, where two modes seemed to be present (*n* = 222) likely due to the increase in sample size. Previous studies have found different modal length frequency distributions for *B. glaciale*, including bimodal (García‐Seoane et al. 2015), multimodal (Hudson et al. 2014) and unimodal (Knutsen et al. 2023). In the Irminger Sea, only a single mode was present in the length distribution of *B. glaciale*, with a large sample size of 3325 individuals (Sigurðsson, Jónsson, and Pálsson 2002). Differences in length frequency distribution modality may be a result of the collection method (gear design and number of samples) causing size selection bias or differences in population demography driven by ontogenetic trends or regional differences in environmental conditions and biotic interactions, such as predation pressure and prey availability (Bax 1998; Ohlberger et al. 2013).

Growth of *B. glaciale* has been estimated in previous studies using the von Bertalanffy growth function (García‐Seoane et al. 2015; Gjösæter 1973; Gjøsæter 1981; Kristoffersen and Salvanes 2009). Along the Norwegian coast, the asymptotic average length values (*L*_∞_) for *B. glaciale* ranged from 64.8 to 83.1 mm, with growth parameters varying between fjords and open ocean regions (Gjösæter 1973; Gjøsæter 1981; Kristoffersen and Salvanes 2009). A more recent study, using an Electronic Length Frequency Analysis (ELEFAN) to estimate von Bertalanffy growth parameters in Norwegian waters using multi‐year data, found *L*_∞_ to be 78.9 mm (Vastenhoud, Mildenberger, et al. 2023). Our current study's estimates were lower than all previous estimates, with a *L*_∞_ of 61.68. Other studies had a wider range of standard lengths of individuals sampled, larger sample sizes and included data over multiple years. These differences between previous results and our study might merely reflect the limitations of our sampling method, which was opportunistic and conducted during a single month and year, as well as differences in gears characteristics. Variation in sampling methods limits the ability to compare population parameters of *B. glaciale* across large oceanographic regions. However, comparisons could be possible if calibration studies between the different methods were to be carried out. Dedicated, large‐scale studies of the mesopelagic are often time‐consuming and costly, but such studies can be facilitated through opportunistic sampling, such as in the current study and through more targeted studies like Knutsen et al. (2023). Standardizing sampling methods across regions would be advantageous, especially with regards to season and sampling gear specificities, as it would allow cross‐regional comparisons and improve our understanding of the biology of various mesopelagic species.

Our study also provides otolith‐fish size relationships that can be used for regional predator dietary studies where these predetermined relationships are essential for accurate back‐calculations and for comparisons across regions in the North Atlantic. Myctophids are the most abundant mesopelagic fish in the North Atlantic and therefore are predated by a range of predators from seabirds to pelagic fishes and cetaceans (Danielsen et al. 2010; Doksæter et al. 2008; Giske et al. 1990; Walkrr and Nichols 1993). Otolith‐fish size relationships for *B. glaciale* exist across the North Atlantic, off the Norwegian and Canadian coasts, and in the Mediterranean Sea (Battaglia et al. 2015; García‐Seoane et al. 2015; Gjösæter 1973; Gjøsæter 1981; Halliday 1970), but have yet to include specimens sampled in our study areas. As found by Gjøsæter (1981), populations in Norway had significant differences in otolith‐fish size relationships between them; thus at even broader spatial scales, it can be expected that these relationships differ.

To conclude, the present study used samples collected in the summer to improve general knowledge on the length and age distributions, length at age relationships, body condition, and growth rate of *B. glaciale* in Icelandic waters. Distinct regional differences in average values of length distribution, age, and body condition suggest possible ontogenetic trends in habitat use, potentially attributed to environmental differences between hydrographic regions, such as temperature, or biotic effects like prey availability. Researching the intricate trophic dynamics of this common prey species requires an understanding of *B. glaciale*'s biology and the relationships between otolith and fish size, which we provide. As this region is anticipated to be subject to environmental changes due to climate change (Astthorsson, Gislason, and Jonsson 2007), understanding factors that may affect the growth and distribution of this high biomass species is especially important in predicting how its role in the ecosystem may be affected. Our study's results coincide with the distribution observed in previous studies (Sigurðsson, Jónsson, and Pálsson 2002) and shows evidence of variability of life history traits of *B. glaciale* between hydrographic regions to the south of Iceland. However, our study represents a snapshot of *B. glaciale* biology and further investigation is necessary to discern the driving factors for these potential differences in the population parameters we observed.

## Author Contributions

**Charlotte S. Matthews:** conceptualization (equal), data curation (equal), formal analysis (lead), investigation (lead), methodology (lead), visualization (lead), writing – original draft (lead), writing – review and editing (equal). **James Kennedy:** conceptualization (equal), data curation (equal), formal analysis (supporting), methodology (equal), supervision (equal), visualization (supporting), writing – review and editing (equal). **Anna H. Ólafsdóttir:** conceptualization (equal), data curation (equal), resources (equal), supervision (equal), validation (equal), writing – review and editing (equal). **Christophe Pampoulie:** conceptualization (equal), data curation (equal), funding acquisition (lead), resources (equal), supervision (equal), writing – original draft (supporting), writing – review and editing (equal).

## Ethics Statement

The fish used in this study were caught during routine monitoring surveys performed by the Marine and Freshwater Research Institute of Iceland and no ethics approval was required.

## Conflicts of Interest

The authors declare no conflicts of interest.

### Open Research Badges

This article has earned an Open Data badge for making publicly available the digitally‐shareable data necessary to reproduce the reported results. The data is available at https://doi.org/10.1594/PANGAEA.930437 and https://doi.pangaea.de/10.1594/PANGAEA.966741.

## Data Availability

All the biological data used in the present study were uploaded in the Pangaea data repository, which can be found under the DOIs: https://doi.org/10.1594/PANGAEA.930437 (Ólafsdóttir and Pampoulie 2020) and https://doi.org/10.1594/PANGAEA.966741 (Matthews et al. 2024).
